# Draft Genome Sequence of the Rhizobacterium *Pseudomonas chlororaphis* PCL1601, Displaying Biocontrol against Soilborne Phytopathogens

**DOI:** 10.1128/genomeA.00130-17

**Published:** 2017-04-06

**Authors:** Carmen Vida, Antonio de Vicente, Francisco M. Cazorla

**Affiliations:** Instituto de Hortofruticultura Subtropical y Mediterránea “La Mayora” Universidad de Málaga, Consejo Superior de Investigaciones Científicas (IHSM-UMA-CSIC), Departamento de Microbiología, Facultad de Ciencias, Universidad de Málaga, Málaga, Spain

## Abstract

In this study, we present the draft genome sequence of the bacterial strain *Pseudomonas chlororaphis* PCL1601. This bacterium was isolated from the rhizosphere of healthy avocado trees and displayed antagonistic and biological control activities against different soilborne phytopathogenic fungi and oomycetes.

## GENOME ANNOUNCEMENT

*Pseudomonas chlororaphis* PCL1601 is a Gram-negative aerobic bacterium isolated from the rhizosphere of a healthy avocado tree allocated in an area affected by avocado white root rot (1), a fungal disease caused by the soilborne phytopathogen *Rosellinia necatrix* (2). The bacterial isolation was carried out from avocado root samples, with further isolation of different nutrient media with cycloheximide (100 µg/ml) to avoid fungal growth interference. *P. chlororaphis* PCL1601 formed opaque and light-yellow colonies when grown on solid nutrient medium, and the colonies were fluorescent when grown in King’s B (KB) medium. Furthermore, PCL1601 presented antagonistic activity against several soilborne pathogens, such as *Fusarium oxysporum* and *Rhizoctonia solani*, but especially to the avocado soilborne pathogens *R. necatrix* and *Phytophthora cinnamomi* (1). Additionally, *P. chlororaphis* PCL1601 showed biological control activity against *R. necatrix* on avocado and to *F. oxysporum* f. sp*. radicis-lycopersici* on tomato (1). This strain is able to produce some antimicrobial compounds, such as hydrogen cyanide (HCN), phenazine-1-carboxylic acid (PCA), and phenazine-1-carboxamide (PCN) (1).

Here, we report the draft genome sequence of *P. chlororaphis* PCL1601. Genomic DNA of *P. chlororaphis* PCL1601 was extracted with the PowerSoil DNA isolation kit (Mo Bio Laboratories, Inc., Carlsbad, CA, USA) after overnight growth in liquid KB medium at 25°C. Genome sequencing was performed at ChunLab, Inc. (Seoul, South Korea) using the Pacific Biosciences 20 K method. Sequencing depth was 223.26× coverage of the genome, which was assembled *de novo* into 25 contigs with the PacBio SMRT Analysis pipeline version 2.3.0 (ChunLab, Inc.). The resulting draft genome sequence was ordered using the genome sequence of *P. chororaphis* PA23 as the template (3). The resulting draft genome sequence was annotated with the NCBI Prokaryotic Genome Annotation Pipeline. Additionally, the secondary metabolite- and antibiotic-encoding gene clusters were predicted with antiSMASH (4).

The draft genome of PCL1601 is 6,755,444 bp in length, containing a G+C content of 64% and 5,897 predicted coding sequences, 17 rRNAs, and 68 tRNAs, features similar to those previously described for the biocontrol strain* P. chlororaphis* PCL1606, also isolated from avocado rhizosphere (5). However, genome annotation displayed a wider range of putative genes involved in general metabolism (carbohydrates, amino acids, lipids, etc.) and transport (such as inorganic ion transport and metabolism, intracellular trafficking, secretion, and vesicular transport). Using antiSMASH, we found 13 potential biosynthetic gene clusters potentially involved in secondary metabolite production, highlighting the phenazine biosynthetic gene cluster, but also bacteriocins (*n* = 4), siderophores (*n* = 2), and nonribosomal peptide synthetases (NRPS; *n* = 2), most of them displaying architecture (higher than 90%) similar to other biosynthetic operons also described in other *P. chlororaphis* strains. The remaining clusters have lower homologies and need further characterization.

### Accession number(s).

This whole-genome shotgun project has been deposited in GenBank under the accession no. MSCT00000000 (from MSCT01000001 to MSCT01000025). The version described in this paper is the first version.
